# Comparing Analytic Methods for Longitudinal GWAS and a Case-Study Evaluating Chemotherapy Course Length in Pediatric AML. A Report from the Children's Oncology Group

**DOI:** 10.3389/fgene.2016.00139

**Published:** 2016-08-05

**Authors:** Marijana Vujkovic, Richard Aplenc, Todd A. Alonzo, Alan S. Gamis, Yimei Li

**Affiliations:** ^1^Division of Oncology, Children's Hospital of PhiladelphiaPhiladelphia, PA, USA; ^2^Department of Preventive Medicine, Keck School of Medicine, University of Southern CaliforniaLos Angeles, CA, USA; ^3^Division of Hematology, Oncology Bone Marrow Transplantation, Children's Mercy Hospitals and ClinicsKansas City, MO, USA

**Keywords:** longitudinal analysis, unrelated population, genome wide association analysis, linear mixed effects model, generalized estimating equations

## Abstract

Regression analysis is commonly used in genome-wide association studies (GWAS) to test genotype-phenotype associations but restricts the phenotype to a single observation for each individual. There is an increasing need for analytic methods for longitudinally collected phenotype data. Several methods have been proposed to perform longitudinal GWAS for family-based studies but few methods are described for unrelated populations. We compared the performance of three statistical approaches for longitudinal GWAS in unrelated subjectes: (1) principal component-based generalized estimating equations (PC-GEE); (2) principal component-based linear mixed effects model (PC-LMEM); (3) kinship coefficient matrix-based linear mixed effects model (KIN-LMEM), in a study of single-nucleotide polymorphisms (SNPs) on the duration of 4 courses of chemotherapy in 624 unrelated children with *de novo* acute myeloid leukemia (AML) genotyped on the Illumina 2.5 M OmniQuad from the COG studies AAML0531 and AAML1031. In this study we observed an exaggerated type I error with PC-GEE in SNPs with minor allele frequencies < 0.05, wheras KIN-LMEM produces more than expected type II errors. PC-MEM showed balanced type I and type II errors for the observed vs. expected *P*-values in comparison to competing approaches. In general, a strong concordance was observed between the *P*-values with the different approaches, in particular among *P* < 0.01 where the between-method AUCs exceed 99%. PC-LMEM accounts for genetic relatedness and correlations among repeated phenotype measures, shows minimal genome-wide inflation of type I errors, and yields high power. We therefore recommend PC-LMEM as a robust analytic approach for GWAS of longitudinal data in unrelated populations.

## Introduction

The development of high-throughput DNA genotyping has accelerated the discovery of single nucleotide polymorphisms (SNPs) associated with common traits and diseases (Welter et al., 2014). Genome-wide analysis (GWAS) accounts for multiple testing and underlying genetic structures but does not capture the natural trajectory of phenotypic traits over time (Nyholt, 2004; Weir et al., 2006). Methodologic challenges for longitudinal GWAS include correlation among phenotypic measurements within an individual and missingness of phenotypic measures over time while accounting for phenomena like genetic relatedness, population stratification, and the influence of stationary or time-varying covariates.

The GWAS Catalog has curated over 2000 cross-sectional studies linked to hundreds of diseases and traits but few longitudinal modeling strategies have been evaluated to date (Kerner et al., 2009; Beyene and Hamid, 2014; Welter et al., 2014). A longitudinal approach to GWAS would be of particular benefit in studying dynamic quantitative traits related to chronic diseases, such as bone mineral density, fasting glucose levels, LDL, and HDL cholesterol, triglycerides, arterial stiffness, and systolic and diastolic blood pressure. When investigating genetic variants on a phenotypic outcome type I errors may occur due to population structure in unrelated populations and/or complex pedigree structure among participants in family based studies. The majority of approaches for the analysis of longitudinal data have emphasized family-based studies in large part in response to various Genetic Analysis Workshops calls for analytic approaches to deal with longitudinal phenotypes in the family-based Framingham Heart Study (FHS). Accounting for pedigree structure usually involves polygenic models incorporating between-individual kinship coefficients in the covariance structure for the random effect in generalized linear models. However, analytic methods for longitudinal GWAS in unrelated individuals are relatively sparse. Furlotte et al. proposed a longitudinal GWAS design that explains phenotypic temporal trends and population structure simultaneously using a kinship coefficient matrix-based linear mixed effects model (KIN-LMEM) (Furlotte et al., 2012). However, it is unconvential to account for population stratification using kinship information in unrelated individuals with exception of a few reports (Kang et al., 2010; Zhang et al., 2010). Typically a GWAS accounts for population-specific variations in allele distribution of SNPs (e.g., population stratification) by including principal components (PCs) as covariates in a linear or logistic regression model (Patterson et al., 2006). Recently, Sikorska et al. used a fast conditional two-step approach based on fitting a linear mixed effects model (LMEM) followed by linear regression as a computationally efficient workaround for LMEM with random intercept and random slope (Sikorska et al., 2015). However, this model overcomplicates scenarios where random slopes are unnecessary.

The field of applied bioinformatics heavily relies on easily reproducible or ready-to-go methods due to restrictions of time, resources, and method complexity. Given the current knowledge gap in longitudinal methods for GWAS in unrelated populations, we present a brief overview of available literature on longitudinal GWAS approaches and compare PC and kinship-based methods to evaluate chemotherapy course length in 2 randomized phase III trials in childhood acute myeloid leukemia (AML). These include: principal component-based generalized estimating equations (PC-GEE), principal component-based linear mixed effects model (PC-LMEM), and kinship coefficient matrix-based linear mixed effects model (KIN-LMEM).

## Materials and methods

### Study design

A total of 624 Caucasian patients were included from two randomized phase III trials of the Children's Oncology Group (COG), AAML0531 (Gamis et al., 2014), and AAML1031 (Meshinchi et al., 2012). These trials enrolled children with newly diagnosed AML using standard chemotherapy in combination with investigations agents. DNA was extracted from remission bone marrow aspirates and genotyped on the Illumina 2.5 M OmniQuad at the Children's Hospital of Philadelphia, PA. The National Cancer Institute's central institutional review board and institutional review boards at each enrolling center approved both studies; patients and their families provided informed consent or assent as appropriate. The trials were conducted in accordance with the Declaration of Helsinki and registered at http://www.clinicaltrials.gov as NCT00372593 (AAML0531) and NCT01371981 (AAML1031).

### Mini review

We aimed to identify all published literature that focused on longitudinal genome-wide association studies (GWAS). Existing publications were identified in PubMed through January of 2016 using the following search terms: “longitudinal,” “repeated measures,” “linear mixed models,” “GWAS,” “genome-wide associations,” and/or “SNP.” Publications not related to longitudinal GWAS were disregarded.

### Phenotype

The primary outcome in the current case-study is chemotherapy course length. Start and end dates of the first 4 courses of chemotherapy were extracted from the COG web portal. Course length was defined as the difference in days between start and end date of each course. The start date was the day the chemotherapy regimen was initiated, and end date was considered as the day that absolute neutrophil counts reached 500/mL and platelets recovered to 20,000/mL in the absence of recurrent or persistent leukemia. The 5th course of chemotherapy on AAML-0531 was excluded from analysis to ensure comparability between the two studies. For quality control purposes course duration was set to missing if the number of course days was < 20 or >100, or a relapse was observed in the respective course. Covariates collected include age, gender, and treatment arm.

### Three longitudinal models

In longitudinal analyses the outcome is a set of repeated measures of course duration over time. We use *Y*_*ij*_ (*i* = 1, …, *n*; *j* = 1, 2, 3, 4) to denote the log-transformed course duration for subject *i* at course *j*. The first model we used is the KIN-LMEM, which is one type of linear mixed effects model (LMEM) and accounts for the individual relatedness by introducing a random effect for each subject that has a covariance matrix estimated from the kinship coefficient (Furlotte et al., 2012). The model assumes that:

(1) $$\begin{array}{l} Y_{ij}=\alpha +\beta \times X_{i}+u_{i}+v_{ij}+\varepsilon_{ij} \end{array}$$

Here *X*_*i*_ represents the state of a particular SNP for subject *i* and is a fixed effect. *u*_*i*_ is the random effect that captures genetic relatedness, and assumed to follow a normal distribution with mean 0, variance σ${}_{\text{u}}^{2}$, and a correlation of *K*_*il*_ between subject *i* and *l* (*K*_*il*_ is the kinship coefficient). *v*_*ij*_ is the random effect for subject *i* and course *j*, which follows a normal distribution with mean 0 and variance σ${}_{v}^{2}$. Note that *v*_*ij*_ for the same subject but different courses are correlated, i.e., cov (*v*_*ij*_, *v*_*ik*_) = σ${}_{v}^{2}$ *D*_*jk*_, but *v*_*ij*_ for different subjects are independent, i.e., cov (*v*_*ij*_, *v*_*lk*_) = 0. ε_*ij*_ is the independent error term and follows a normal distribution with mean 0 and variance σ^2^. The three types of random effects *u*_*i*_ *v*_*ij*_ and ε_*ij*_ are assumed to be independent. This model implies that the outcomes for the same subject at different courses have a correlation:

$$\begin{array}{l} \text{Corr}\left(Y_{ij},Y_{ik}\right)=\;\left(\sigma_{v}^{2}D_{jk}+\sigma_{u}^{2}\right)\;∕\;\left(\sigma^{2}+\sigma_{v}^{2}+\sigma {\;}_{u}^{2}\right); \end{array}$$

and the outcomes for two different subjects at any course are also correlated due to their genetic relatedness:

$$\begin{array}{l} \mathrm{Corr}\left(Y_{ij},Y_{lk}\right)=\sigma_{u}^{2}K_{il}\;∕\;\left(\sigma^{2}+\sigma_{v}^{2}+\sigma_{u}^{2}\right) \end{array}$$

The second model we used is PC-LMEM, a LMEM that accounts for individual relatedness through the use of principal components (PCs) as covariates in the model to adjust for population structure, a common practice in unrelated populations.

The model assumes that:

(2) $$\begin{array}{lll} Y_{ij} & = & \alpha +\beta \times X_{i}+\gamma \times Z_{ij}+\delta_{1}\times P_{1i}+\;\delta_{2}\times P_{2i}\\ \;+\delta_{3}\times P_{3i}+v_{i}+\varepsilon_{ij} \end{array}$$

Again *X*_*i*_ is the SNP for subject *i* as a fixed effect. *Z*_*ij*_ is the fixed effect for course. Rather than using a random effect *u*_*i*_ to account for genetic relatedness as in the KIN-LMEM, the PC-LMEM uses the principle components as covariates (three PCs here: *P*_1*i*_, *P*_2*i*_, and *P*_3*i*_). *v*_*i*_ is the random effect to reflect the correlation among the repeated measures within a subject, but takes a simple form of a random intercept, and is assumed to be independent (i.e., cov (*v*_*i*_, *v*_*l*_) = 0) and normally distributed (mean 0, variance σ${}_{v}^{2}$). ε_*ij*_ is the independent error term following normal distribution with mean 0 and variance σ^2^, and the two types of random effects *v*_*i*_ and ε_*ij*_ are assumed to be independent. This model implies that the outcomes for the same subject at different courses have a correlation:

$$\begin{array}{l} \text{Corr }\left(Y_{ij},\;Y_{ik}\right)=\sigma_{v}^{2}\;∕\;\left(\sigma^{2}+\sigma_{v}^{2}\right); \end{array}$$

but the outcomes for two different subjects at any course are independent since we already adjusted for population substructure through PCs:

$$\begin{array}{l} \text{Corr }\left(Y_{ij},Y_{lk}\right)=0. \end{array}$$

PC-GEE is the third model, an alternative longitudinal model to LMEM. This model adjusts for individual relatedness through the use of PCs and accounts for correlations of repeated measures within a subject through the use of robust covariance structure. Specifically the model assumes that:

(3) $$\begin{array}{l} Y_{ij}=\alpha +\beta \times X_{i}+\gamma \times Z_{ij}+\delta_{1}\times P_{1i}+\delta_{2}\times P_{2i}+\delta_{3}\times P_{3i}+\varepsilon_{ij} \end{array}$$

$$\begin{array}{l} \text{Var}\left(Y_{ij}\right)=\sigma^{2},\;\mathrm{Corr}\left(Y_{ij},Y_{ik}\right)=\rho ,\mathrm{Corr}\left(Y_{ij},Y_{lk}\right)=0 \end{array}$$

The notations here are the same as in Equation (2), except that we no longer have the random effect *v*_*i*_ and rather explicitly specify an “exchangeable” correlation structure ρ for the repeated measures within a subject. We note that LMEMs (KIN-LMEM and PC-LMEM) are likelihood-based and valid under the missing at random (MAR) assumption, but PC-GEE is valid only under the missing completely at random (MCAR) assumption.

### Statistical methods

Extensive genotyping quality control checks were performed using PLINK, and SNPs were excluded from analysis in case of (1) call rates < 95%; (2) monomorphic SNPs (MAF < 0.01); and (3) deviation from Hardy-Weinberg Equilibrium (*P* < 10^−5^) (Purcell et al., 2007). GCTA software was used to identify duplicates among genotyped samples, calculate the kinship coefficients matrix, and ancestral groups were constructed via Principal Components Analysis (PCA), and patients showing familial structure and/or cryptic relatedness were excluded (Yang et al., 2011). The genotype data of the subset of unrelated patients in our cohort was then merged with data from Caucasian participants from HapMap3. Naive Bayes classification was performed using HapMap3 as the training set. Remaining heterogeneity between individuals of European descent are illustrated with scatterplots between principal components. Longitudinal analyses were performed with R packages gee and lme4, and the R script provided by Furlotte et al. (2012). Covariates in PC-GEE and PC-LMEM include course number and seven principal components as indicators of ancestry.

We used several metrics to evaluate the performance of the three analytics methods. First, quantile-quantile (Q-Q) plots were estimated with the R package qq-man to ensure that the observed *P*-value distribution follows a Chi-Square null distribution with exception of the extreme tail. Second, Manhattan plots were generated to evaluate the differences in the global pattern of the significance across three methods. Third, correlation coefficients between the observed *P*-values from different methods were calculated to examine their concordance. Finally, we test the interchangeability of the results by evaluating to what extent the different methods produce similar *P*-values, taken at decreasing thresholds of 0.5, 0.1, 0.01, 0.001, and 0.0001. We then summarized the predictive accuracy for each method by their competing alternatives by calculating true positive and false positive rates using the ROCR package in R. We plotted all receiver operating characteristic (ROC) curves considering the area under the curve (AUC) as a measure of predictive performance.

## Results

Our search for studies related to longitudinal GWAS yielded 19 results. Table 1 summarizes longitudinal GWAS evaluation studies to date. The majority of the study focused on methods accounting for complex pedigree structure structures. Nine studies performed a single-step longitudinal analysis where LMEM and GEE were mostly used (Chang et al., 2009; Kerner et al., 2009; Park et al., 2009; Zhu et al., 2009; Furlotte et al., 2012; Choi et al., 2014; Hossain and Beyene, 2014; Tan et al., 2014). A two-step approach was utilized by others where a first a summary measure for the longitudinal phenotype was extracted and subsequently single observational analysis was performed (Aulchenko et al., 2007; Fradin and Fallin, 2009; Roslin et al., 2009; Yan et al., 2009; Eu-Ahsunthornwattana et al., 2014; Musolf et al., 2014; Vaitsiakhovich et al., 2014; Wang et al., 2014; Xia and Lin, 2014). Incidental reports include cluster analysis, hierarchical multi-level linear modeling, Bayesian LASSO with B-splines, MLGM, and MASAL using various software packages. Model selection, missing data and small sample size were the two most common challenges. There were no loci consistently found the across studies. Linear mixed models were most accurate at confirming known SNPs.

**Table 1:** **Literature overview of longitudinal GWAS analysis**.

**References**	**Design**	**Outcome**	**Covariates**	**Familial relatedness**	**Missingness**	**Analysis**	**Software**
Sikorska et al., 2015	Family	Linear	Time-varying	NA	MAR/MCAR	Reduced conditional LMEM + GWAS on random slopes	R
Choi et al., 2014	Family	Binary	Stationary	Random effects	MAR	GEE, family-specific GLMEM	PLINK, SAS, R
Eu-Ahsunthornwattana et al., 2014	Family, unrelated	Linear	Stationary	Variance-covariance structure	MAR	PHENO LIN REG, GWAS on residuals	PLINK, EMMAX, FaST-LMEM, GenABEL
Hossain and Beyene, 2014	Family	Linear	Stationary	Variance-covariance structure	MAR	LMEM	R
Musolf et al., 2014	Family, unrelated	Linear	Time-varying	(Q)TDT	NA	Cluster analysis, GWAS	SAS, TDT-HET
Tan et al., 2014	Family	Linear	Stationary	Kinship matrix	NA	Two-level hierarchical linear model with random coefficients	
Vaitsiakhovich et al., 2014	Unrelated	Delta	Stationary	NA	MAR, MCAR, MNAR + imputation	GWAS on mean change of imputed data	INTERSNP
Wang et al., 2014	Family	Linear	Stationary	NA	MAR + imputation	LMEM and a two-stage approach: random intercept model + GWAS	R, PLINK
Xia and Lin, 2014	Family	Binary	Stationary	Inbreeding coefficient in Bayesian	MAR	Logistic Bayesian LASSO, B-spline, partial GLMEM	R, Hapassoc
Furlotte et al., 2012	Unrelated	Linear	NA	Variance-covariance structure	NA	Modified LMEM	R
Chang et al., 2009	NA	Binary	Time-varying	Random effects	NA	GMM	SAS
Fradin and Fallin, 2009	Family	Binary	Stationary	NA	NA	LOG REG, conditional on risk set	SAS
Kerner et al., 2009	Family	Linear	Stationary	Random effects	NA	GMM, QTL analysis	Mplus, Goldenhelix
Luan et al., 2009	Family	Linear	Stationary	Random effects	NA	LMEM	Stata, SAS
Park et al., 2009	Family	Binary	Stationary	Pedigree membership as covariate	NA	GEE	SAS
Roslin et al., 2009	Unrelated	Linear	Stationary	NA	NA	MLGM, LIN REG	Mplus, PLINK
Yan et al., 2009	Unrelated	Binary	Stationary	NA	NA	PAF	SAS
Zhu et al., 2009	Family	Linear	Time-varying	Sibship group membership	NA	MASAL	MASAL
Aulchenko et al., 2007	Family	Linear	NA	Random effects	NA	GWAS on LMEM phenotype residuals	GRAMMAR

*GEE, generalized estimating equation; GLMEM, generalized linear mixed model; GRAMMAR, genome-wide rapid association using mixed model and regression; GWAS, genome-wide association studies; HWE, Hardy-Weinberg equilibrium; LASSO, least absolute shrinkage and selection operator; LMEM, linear mixed model; MAF, minor allele frequency; MCAR, missing completely at random; MGR, missing genotype rate; MNAR, missing not at random; PH, proportional hazard; SNP, single-nucleotide polymorphism; GMM, growth mixture modeling; QTL, quantitative trait locus; LMEM, linear mixed modeling; GEE, generalized estimating equations; MLGM, multivariate linear growth modeling; PAF, population attributable risk fraction; MASAL, multivariate adaptive splines for the analysis of longitudinal data*.

**Figure 1:**
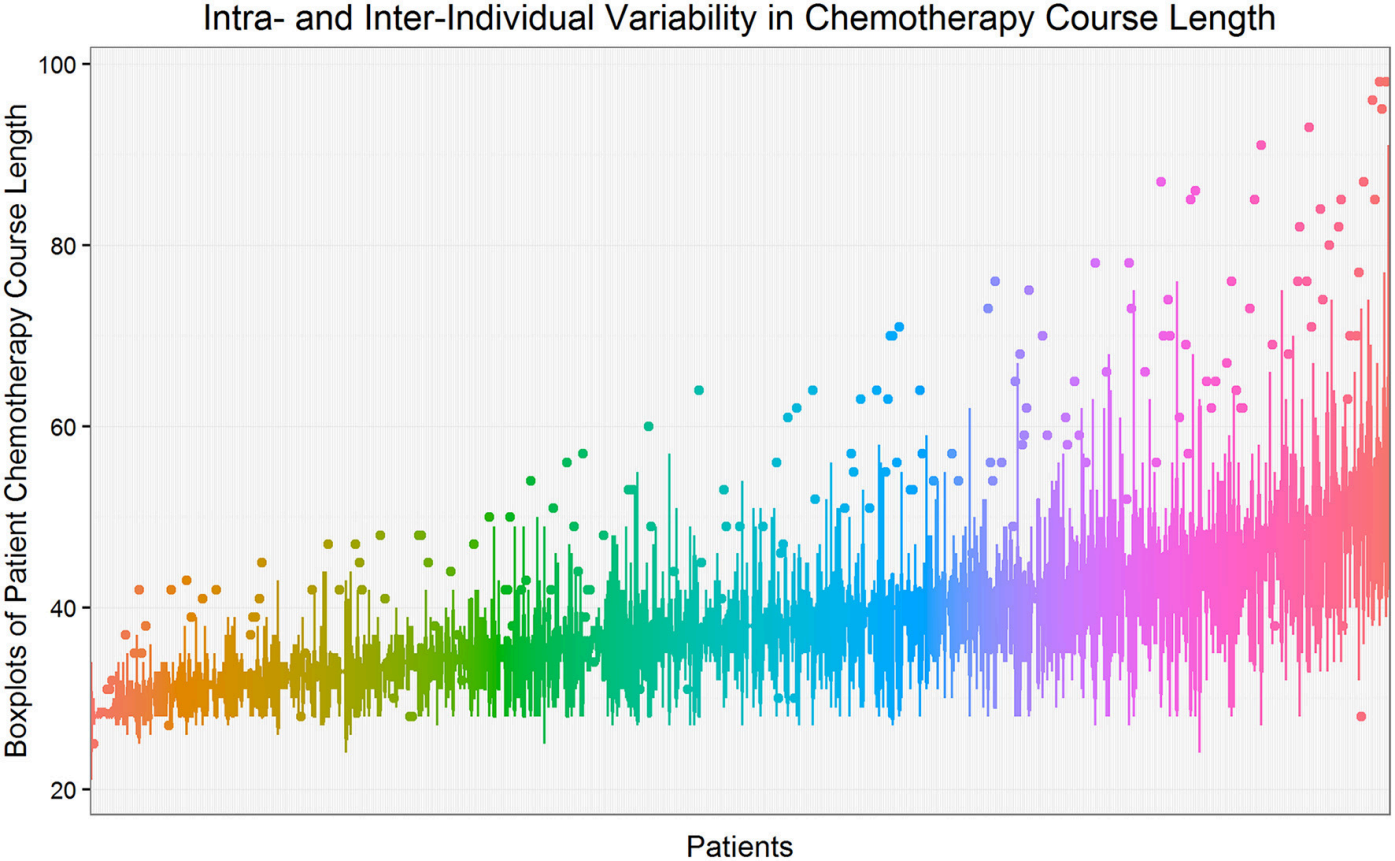
**Intra- and inter-individual variability in chemotherapy course length**. Each line represents a patient's median and interquartile range (IQR), sorted from the lowest to the highest median value. The overall median chemotherapy course length is 36 days (IQR 32–42 days). Course length observations less than −1.5^*^IQR below 25^th^ percentile or more than 1.5^*^IQR above the 75^th^ percentile are considered to be outliers and are shown as isolated points.

**Figure 2:**
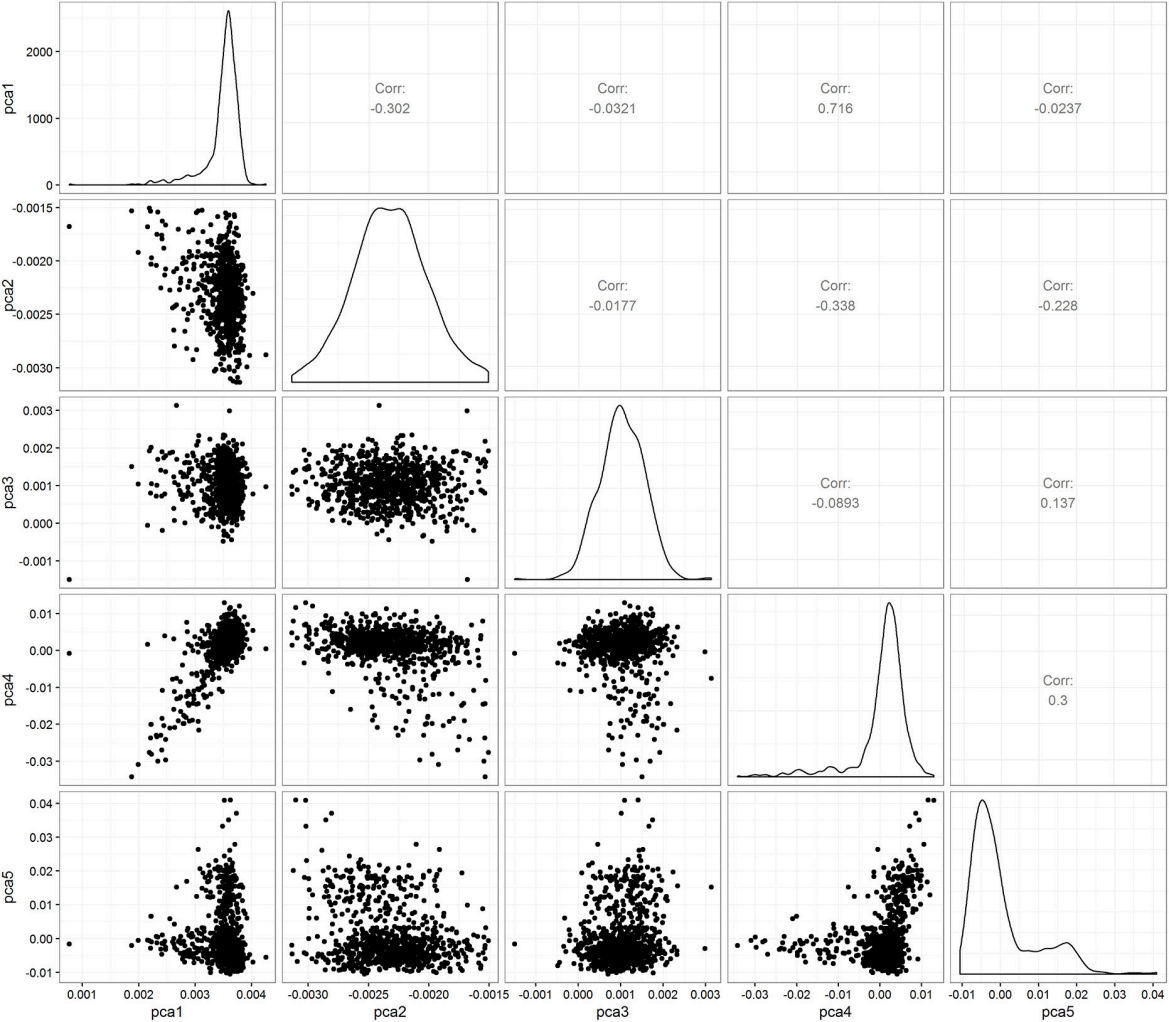
**Pairwise PC plots within 624 Caucasian patients with AML**.

Figure 1 shows the intra- and inter-individual variability in chemotherapy course length for the cohort of 624 pediatric AML patients. Each line represents a patient's median and interquartile range (IQR), sorted from the lowest to the highest median value. The overall median chemotherapy course length is 36 days (IQR 32–42 days). The ancestry plot depicting the relationship between principal components (Figure 2) illustrates the within-population heterogeneity for all patients of European descent.

**Figure 3:**
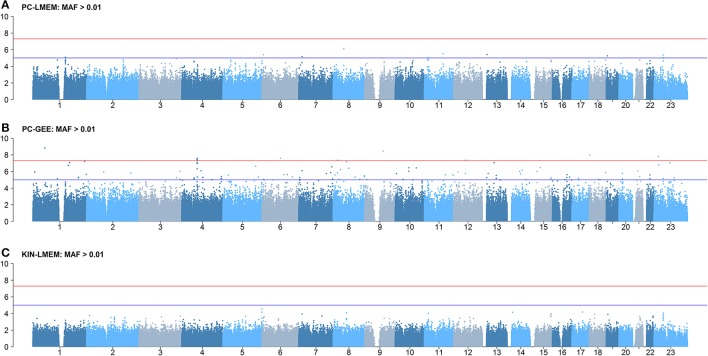
**Results of longitudinal GWAS analyses on three different methods**. **(A)** PC-LMEM: MAF > 0.01; **(B)** PC-GEE: MAF > 0.01; **(C)** KIN-LMEM: MAF > 0.01.

**Figure 4:**
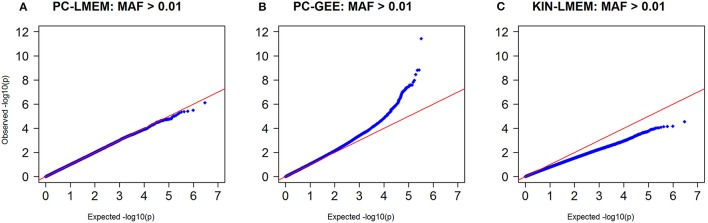
**Q-Q plots of ***P***-values from three longitudinal GWAS analyses**. **(A)** PC-LMEM: MAF > 0.01; **(B)** PC-GEE: MAF > 0.01; **(C)** KIN-LMEM: MAF > 0.01.

Figures 3, 4 show the Manhattan and QQ-plots for PC-GEE, PC-LMEM, and KIN-LMEM. The QQ-plot from PC-GEE shows an increased rate of false positives indicating the necessity for an additional round of genomic control. The KIN-LMEM model suffers of a substantial type II error rate under the null Chi Square distribution. With KIN-LMEM a small cluster of SNPs of below genome-wide threshold *P*-values was observed on chromosome 5, which was not captured with PC-GEE and PC-LMEM. Finally, PC-LMEM shows an expected pattern of significance with low type I and type II error rates.

**Figure 5:**
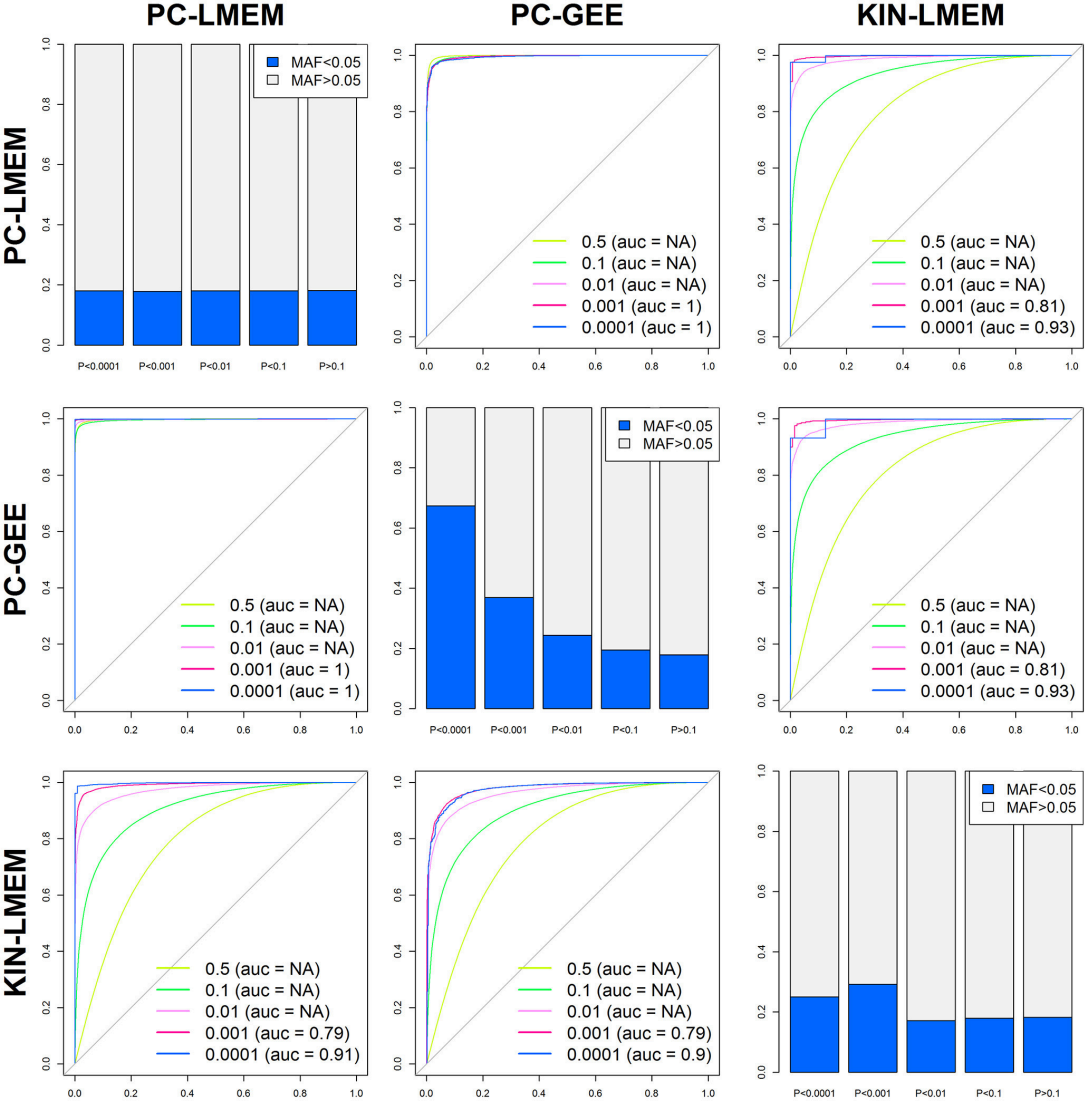
**Between method accuracy and rare variant distribution across ***P***-values**. The 6 off-diagonal ROC curves represent the accuracy of each method predicting different *P*-value percentiles of competing methods (*P* < 0.5, *P* < 0.1, *P* < 0.01, *P* < 0.001, and *P* < 0.0001). The diagonals show the relative distribution of rare SNPs (MAF < 0.05) for various *P*-value cut-offs of each method (e.g., *P* > 0.1, 0.01 < *P* < 0.1, 0.001 < *P* < 0.01, 0.0001 < *P* < 0.001, and *P* < 0.0001).

Even though the overall *P*-values show distinct global patterns as evident from the QQ-plot and Manhattan plot, the three methods show a strong underlying comparability as measured by correlation coefficients. The correlation coefficient between PC-LMEM and PC-GEE *P*-values exceeds > 0.99. The correlation between PC-LMEM and KIN-LMEM is relatively high (*r*^2^ = 0.605), and the same accounts for PC-GEE and KIN-LMEM (*r*^2^ = 0.603). Further exploration of between-method similarities and differences are shown in Figure 5. The diagonals in this figure show the relative distribution of rare SNPs (MAF < 0.05) for various *P*-value cut-offs of each method (e.g., *P* > 0.1, 0.01 < *P* < 0.1, 0.001 < *P* < 0.01, 0.0001 < *P* < 0.001, and *P* < 0.0001). In PC-GEE the lowest *P*-values (*p* < 0.001) are enriched for rare SNPs. In contrast, PC-LMEM and KIN-LMEM analyses are less affected by SNP prevalance. Off-diagonal plots show ROC curves with AUCs for the prediction of *P*-value thresholds of the respective method by its competing approaches. The results from PC-LMEM can be predicted with very high accuracy by PC-GEE across the *P*-value spectrum (AUC > 0.99 for each *P*-value threshold), which is in line of expectation due to their extremely high correlation. KIN-LMEM *P*-values show a more heterogeneous range of predictive performances by both PC-LMEM and PC-GEE, where improvements in predictive accuracy are observed with decreasing *P*-values. For example, the AUC exceeds 0.99 for the lower range of KIN-LMEM *P*-values (e.g., *p* < 0.01). In summary, any observed *P*-value < 0.001 from each method shows to be very accurately predicted by it's competing methods (AUC > 0.99).

**Figure 6:**
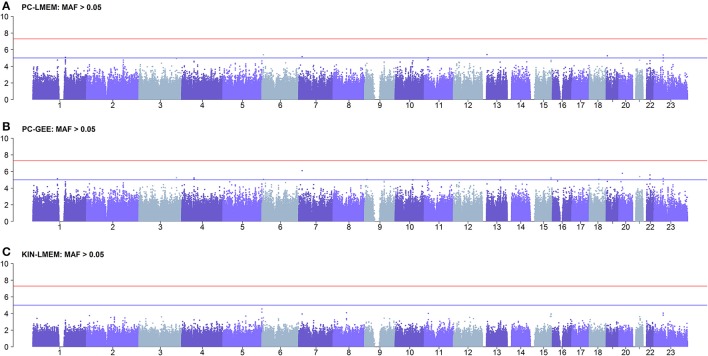
**Results of longitudinal GWAS analyses on three different methods where MAF > 0.05**. SNPs with MAF < 0.05 have been excluded. **(A)** PC-LMEM: MAF > 0.05; **(B)** PC-GEE: MAF > 0.05; **(C)** KIN-LMEM: MAF > 0.05.

**Figure 7:**
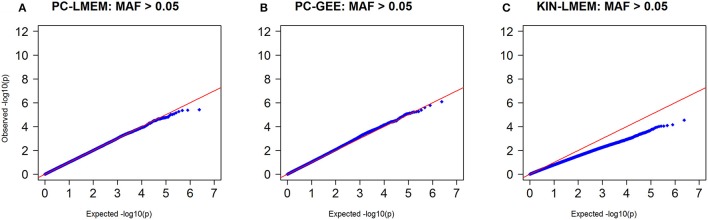
**Q-Q plots of ***P***-values from three longitudinal GWA analyses where MAF > 0.05**. SNPs with MAF < 0.05 have been excluded. **(A)** PC-LMEM: MAF > 0.05; **(B)** PC-GEE: MAF > 0.05; **(C)** KIN-LMEM: MAF > 0.05.

To confirm whether the inflated type I error in PC-GEE is attributable to rare variants, we recreated the Manhattan and QQ-plots excluding SNPs with MAF < 0.05 (Figures 6, 7). The QQ-plots for PC-LMEM and KIN-LMM were not affected, however the inflated false positive rate in PC-GEE has normalized and observed *P*-values follow a Chi-Square null distribution. These results indicate that longitudinal GWAS using GEE is not an appropriate method for investigating rare variant associations. In the longitudinal GWAS analyses restricted to MAF > 0.05, PC-GEE, and PC-LMM show almost perfect concordance.

## Discussion

We compared the performance between three models for GWAS data with a longitudinal, repeatedly measured outcome with missing data from two Phase III clinical trials. The three methods show remarkably different rates of false positives and false negatives under the Chi-Square null distribution. Our results indicate that the PC-LMEM approach shows the better performance to KIN-LMEM and PC-GEE in this scenario of unbalanced longitudinal data in an unrelated population. Modeling longitudinal data differs from single observations because it needs to account for correlation structures arising from repeated measurements of a trait within an individual, and potential differential missingness of repeated phenotype measures over time.

Two methods to account for genetic relatedness between study individuals are adjustment of PC as covariates (as the PC-LMEM) and the use of a kinship matrix (as the KIN-LMEM). PC-LMEM accounts for population substructure by restriction and genomic control, e.g., first identifying close relatives to remove them from analysis, then correcting for broad sample structure using principal components or spatial information and finally correcting for the residual inflation with genomic control.

The KIN-LMEM method is used in complex pedigrees encompassing both hidden relatedness and population stratification. Recently, linear mixed models based approaches have been proposed as an alternative to PC-based approaches when adjusting for population stratification in studies of unrelated individuals (Kang et al., 2010; Zhang et al., 2010). The covariance structure for the random effect is generally assumed to correspond to that implied by a polygenic model, incorporating the genetic relationship (kinship) between each pair of individuals. The rationale for this approach is that apparently unrelated individuals may nevertheless display distant levels of common ancestry. In our study it seems the KIN-LMEM inflates the type II error rates which may lead to a loss a power and the inability to detect true positives. However, for longitudinal GWAS in family studies, PC-based methods may be insufficient to account for more complex genetic relatedness structures, and KIN-LMEM or other family-based approaches should be considered.

Our observations indicate that GEE is unable to produce a reliable association in SNPs with a low MAF. Even though GEE does not require assumptions on the joint distribution of observed data and random effects it does depend on a correct specification of covariate-specific means of the outcome. In rare variants the minor allele group contains to few observations to produce reliable marginal population means (Diggle, 2002).

In our study we used chemotherapy course as a categorical covariate and therefor did not use Sikorska method. Sikorska's fast two-step estimation method is recommended in a scenario of continuous time elements and both random intercepts and random slopes are needed. If time (e.g., chemotherapy course) should be considered as categorical and thus random slope is not applicable, using our method is more appropriate. A limitation of our study is the lack of a true genetic association with course duration that inhibits the generation of false negative rates and operational characteristics for comparing the detection of SNPs truly associated with the outcome.

In pediatric AML, chemotherapy course length is a primary determinant of infection risk and shows a substantial intra-individual correlation and inter-individual variability. We previously showed that MTHFR polymorphisms were not associated with a significantly altered risk of chemotherapy course length (Aplenc et al., 2005). However, Murphy et al. showed that in the MTHFR C677CT wiltypes, folic acid was significantly associated with more than two-fold increased neutrophil recovery compared to the CT and TT genotypes (Murphy et al., 2012). Unraveling genetic components for chemotherapy course length has the clinical potential for personalized toxicity monitoring. We anticipate on reanalyzing the data with a larger sample size in future using PC-LMEM.

In conclusion, the false positive rates of the three methods were remarkably different and the PC-LMEM model seems to provide a reliable approximation of the *P*-value for the effect of SNP on a temporal phenotype. PC-LMEM loses no power compared to the KIN-LEMM and shows no inflation of the type II error rate compared to PC-GEE in SNPs with low MAFs. PC-LMEM hold perhaps the greatest promise for longitudinal GWAS because of its flexibility in accounting for correlation structures and its validity for rare variant association studies. We recommend a practical framework based on PC-LMEM for high-throughput genetic analysis of longitudinal data implemented in a PLINK/R framework in a high-performance computing environment.

## Ethics statement

Written informed consent and ethics committee approval were available for all study participants. Informed consent was obtained in accordance with the Declaration of Helsinki. The institutional review boards of all participating institutions approved the clinical protocol, and the COG Myeloid Disease Biology Committee approved all studies.

## Author contributions

The authors have been substantial contributions to (1) the conception and design of the study, (2) acquisition of data (MV, TA, AG, RA), (3) analysis and interpretation of data (MV, RA, YL), (4) drafting the article or revising it critically for important intellectual content (MV, YL, RA, TA, AG), and (5) final approval of the version to be submitted (MV, YL, RA, TA, AG).

## Funding

Research is supported by the Chair's Grant U10 CA98543-08 and Statistics and Data Center Grant U10 CA180899-02 of the Children's Oncology Group, and Dr. Aplenc's R01 grant 1R01 CA165277, from the National Institutes of Health (NIH). The content is solely the responsibility of the authors and does not necessarily represent the official views of the NIH.

### Conflict of interest statement

The authors declare that the research was conducted in the absence of any commercial or financial relationships that could be construed as a potential conflict of interest. The reviewer FS and handling Editor declared their shared affiliation, and the handling Editor states that the process nevertheless met the standards of a fair and objective review.
